# An eHealth intervention (ManGuard) to reduce cardiovascular disease risk in male taxi drivers: protocol for a feasibility randomised controlled trial

**DOI:** 10.1186/s40814-022-01163-4

**Published:** 2022-09-14

**Authors:** James McMahon, David R. Thompson, Kevin Brazil, Chantal F. Ski

**Affiliations:** 1 School of Nursing and Midwifery, Queen’s University Belfast, 97 Lisburn Road, Belfast, BT9 7BL UK; 2 Integrated Care Academy, University of Suffolk, 19 Neptune Quay, Ipswich, IP4 1QJ Ipswich, UK

**Keywords:** Cardiovascular diseases, Risk, Telemedicine, Men, Randomised controlled trial as topic

## Abstract

**Background:**

Men are at higher risk then women of developing cardiovascular disease (CVD), and male taxi drivers are a particularly high-risk group because of their typically unhealthy behaviours, such as poor eating habits, smoking and sedentary lifestyle. However, only two studies of behavioural interventions targeting taxi drivers have been identified, one of which reported a high attrition rate. Therefore, an eHealth intervention co-designed by taxi drivers may prove more acceptable and effective. The aim of this study is to assess the feasibility an eHealth intervention (ManGuard) to reduce CVD risk in male taxi drivers.

**Methods:**

A randomised wait-list controlled trial will be conducted with a sample of 30 male taxi drivers to establish feasibility, including recruitment, engagement, and retention rates. Program usability and participant satisfaction will be assessed by a survey completed by all participants at 3 months after allocation. Additionally, an in-depth qualitative process evaluation to explore acceptability of the intervention will be conducted with a subset of participants by semi-structured telephone interviews. Preliminary efficacy of ManGuard for improving key CVD-related outcomes will be assessed, including biomarkers (total cholesterol, HDL cholesterol, LDL cholesterol, triglycerides, and total/HDL cholesterol ratio), blood pressure, anthropometry (body mass index, body fat percentage, and waist circumference), physical activity (accelerometery, and self-report) and psychosocial status (health-related quality of life, self-efficacy, and social support). Outcomes will be assessed at baseline, 7 weeks, and 3 months after group allocation. The wait-list control group will be offered access to the intervention at the completion of data collection.

**Discussion:**

eHealth interventions show potential for promoting behaviour change and reducing CVD risk in men, yet there remains a paucity of robust evidence pertaining to male taxi drivers, classified as a high-risk group. This study uses a randomised controlled trial to assess the feasibility of ManGuard for reducing CVD risk in male taxi drivers. It is envisaged that this study will inform a fully powered trial that will determine the effectiveness of eHealth interventions for this high risk and underserved population.

**Trial registration:**

This trial has been registered prospectively on the ISRCTN registry on 5 January 2022, registration number ISRCTN29693943

**Supplementary Information:**

The online version contains supplementary material available at 10.1186/s40814-022-01163-4.

## Background

Cardiovascular disease (CVD), cancer, chronic pulmonary disease, and diabetes account for approximately 80% of non-communicable disease (NCD) related deaths and develop as the result of a combination of genetic, physiological, environmental, and behavioural factors [74]. However, behavioural risk factors such as tobacco use, excessive alcohol consumption, poor dietary choices, and physical inactivity, are most associated with the development of NCDs [31].

CVD remains the leading cause of NCD-related mortality worldwide, accounting for one-third of all deaths in 2019 [75]. Males are at a higher risk of developing CVD than females throughout their lifetime until older age [73]. In the United Kingdom (UK), including Northern Ireland, where 12% of the population are living with CVD and 30% of CVD-related deaths will be premature [8], men have a higher incidence of myocardial infarction and of diabetes.

### Taxi driving and CVD risk

Taxi driving is a male dominated occupation [53] and is associated with an increased risk of CVD [13]. Factors include long working hours and traffic congestion, causing prolonged psychological stress [13, 70], as well as sleep deficiency, irregular work patterns, noise, and regular exposure to air pollution [76]. There is a higher prevalence of modifiable CVD-related risk factors and behaviours among taxi drivers, including having high blood pressure, being overweight or obese, lacking fruit and vegetables, smoking, consuming excessive alcohol and spending long periods sedentary [4, 13, 17, 22, 70, 76].

The long periods of sedentary behaviour and subsequent lack of physical activity directly affect the development of CVD [66], associated with elevated blood pressure, weight gain and poor cholesterol profiles in taxi drivers [3, 12]. Physical activity can mitigate such adverse effects in this population [67], for example, by protecting against hypertension and counter-balancing the negative impact of age, working hours, and weight [69].

Despite the increased risk of CVD and high prevalence of CVD-related risk factors in taxi drivers [3–5, 13, 22, 23, 27, 48, 51, 54, 69], only two studies of behaviour change interventions targeted at this group have been identified [14, 23]. Both interventions were aimed at increasing physical activity through walking, assessing steps [14, 23], days walked, and amount of time walked [14]. Improvements in physcial activity were observed for participants receiving either intervention, with signficant improvements observed for all walking-related measures in one [14]. However, a major limitation to the study by Gany et al. [23] was the high rate of participant attrition (61%). To improve engagement and reduce attrition of men in health behaviour change interventions, a gender-sensitised approach to development is recommended [7, 55].

### eHealth interventions for behaviour change

eHealth offers a means of tailoring interventions to meet the needs of end-users and has the potential to improve intervention uptake, adherence, and success [65]. Defined as “the use of technology to improve health, well-being and healthcare” [65], eHealth provides participants with an easily accessible and easy to use intervention [45]. The ease of access and use may be of benefit to taxi drivers, presenting as a hard-to-reach group due to the remote nature of the occupation. A recent systematic review and meta-analysis reported the success of eHealth interventions in promoting behaviour change among those living with NCDs [18]. Another recent systematic review and meta-analysis by us found similar findings for the reduction in CVD risk in men, with improvements in body weight, body mass index (BMI), waist circumference and blood pressure observed as a result of eHealth interventions [39].

However, weaknesses of eHealth interventions include poor adherence and high rates of attrition [33, 35]. To counter this, it is important that consideration is given to the needs of the end-users, iteratively developed, grounded in behaviour change theory and rigorously evaluated prior to the dissemination of findings [46, 50, 65].

The IDEAS (Integrate, Design, Assess, and Share) framework has been proposed for the design, development, and evaluation of eHealth interventions [46], and was utilised for the development of ManGuard. This 10-phase framework draws on the strengths of several approaches for eHealth interventions including design thinking, user-centred design, behaviour change theories, evaluation, and dissemination [46]. The framework recommends an intervention be pilot tested to assess preliminary efficacy, providing an opportunity to refine the study protocol prior to progressing to a definitive randomised controlled trial [46].

Due to greater levels of engagement in CVD-related risk behaviours, behaviour change interventions could prove beneficial for this high-risk group. However, little appears to have been done to help reduce the observed risk, with significant participant attrition observed in one of the two trials studying this population. In particular, the provision of an eHealth intervention may prove beneficial due to the ease of access and use for a hard-to-reach population. Guided by the IDEAS framework, a novel eHealth intervention (ManGuard) has been developed alongside end-users through an iterative process, ensuring program acceptability and user satisfaction. This paper describes the study protocol for a feasibility randomised controlled trial to pilot test ManGuard, an eHealth intervention that aims to reduce CVD risk in male taxi drivers in Northern Ireland.

### Research aims and objectives

This study aims to assess the feasibility of ManGuard. Study objectives are the following:

- Primary—to assess:
  - ◦ Acceptability: recruitment, retention, and engagement rates
  - ◦ Program usability and participant satisfaction: process evaluation and survey
- Secondary—to assess the preliminary efficacy of ManGuard compared to usual care in improving the following outcomes:
  - ◦ Clinical indices: CVD biomarkers (cholesterol, glucose), blood pressure, anthropometry (BMI, waist circumference, body fat percentage)
  - ◦ Physical activity
  - ◦ Psychosocial status: health related quality of life, self-efficacy, social support

## Methods

### Study design

The study will be a randomised wait-list controlled trial followed by a process evaluation. A wait-list control is employed to increase acceptability of the research protocol to the participants in the control group, and thereby to decrease attrition effects [16]. Both groups will be matched on participant demographics, with those in the wait-list control group receiving the intervention following the final data collection at 3 months after allocation. Semi-structured telephone interviews will be conducted at 3 months as part of a process evaluation.

### Research setting

The research will be conducted in a community setting with male taxi drivers working in Northern Ireland.

### Participants

#### Eligibility criteria for participants

- Adult males (≥ 18 years)
- Working part-time or full-time as a taxi driver in Northern Ireland
- Having access to the internet and a smartphone that allows for the downloading of mobile applications.

As this is a feasibility randomised controlled trial, no formal power calculation will be completed to determine sample size. Recommendations on the appropriate sample size for pilot and feasibility studies vary greatly, with sample sizes ranging between 24 and 50 participants [57]. We have chosen a sample size of 30 for this study, as recommended by the National Institute for Health Research (NIHR) in the UK [30].

### Recruitment

As a result of taxi drivers working both independently and for large companies, recruitment will be conducted via the online social media platform ‘Facebook’ to increase reach. This method of recruitment was employed previously for the recruitment of participants to aid in the co-design of the ManGuard intervention. A Microsoft Forms link, with a brief description of the study, will be posted into two separate Facebook groups dedicated to taxi drivers working across Northern Ireland, estimated as having 4200 members. The link will be posted weekly, with recruitment open for a duration of 17 months, ceasing once the target sample has been achieved. Those who are interested will provide their name and email address, with the lead researcher then sending a participant information sheet with the inclusion/exclusion criteria.

Those that meet the inclusion criteria will be invited to the Northern Ireland Clinical Research Facility (NICRF) to be formally recruited to the study and complete baseline testing. During the participants’ final visit to the NICRF, participants in the intervention group will be offered the opportunity to participate in the semi-structured telephone interviews as part of a process evaluation. Verbal consent will be obtained and recorded at the commencement of the telephone interview, with participants made aware of their right to withdraw from the interview at any time.

### Randomisation

Participants will be randomly allocated by blocked randomisation to the intervention and wait-list control group using the Statistical Package for the Social Sciences (SPSS V.24). Due to the small sample size, blocked randomisation is deemed an appropriate technique for reducing the risk of bias and ensuring equal allocation between groups [20]. The wait-list control group will not be provided with any materials relating to the intervention or recommendations on how they can improve on their current lifestyle habits to maintain a ‘true control’. Upon the completion of data collection at 3 months, participants in the wait-list control group will be offered access to the intervention. The randomisation procedure will be carried out by a researcher not affiliated with the study. Participant allocation will be placed in a sealed envelope and opened by the lead researcher upon completion of baseline data collection.

### Intervention

The intervention is an eHealth program (ManGuard) that aims to reduce CVD risk in male taxi drivers. ManGuard was developed utilising a co-design approach, involving an ongoing collaboration between the research team, a computer scientist, and end-users (taxi drivers). Utilising co-design with an iterative approach is crucial for the success of an eHealth intervention [64]. First, an online survey was disseminated to gain an insight into life as a taxi driver in Northern Ireland and to guide the initial development of ManGuard. Utilising the iterative process between a Project Advisory Group (PAG) and focus groups with end-users, low-fidelity prototypes for the program and materials that would make up the ‘content’ where refined. The inclusion of behaviour change theory in the development of health interventions is reported as an essential component for improving their effectiveness [59]. The Behaviour Change Wheel (BCW) was incorporated throughout the development of ManGuard, providing a pragmatic framework and ensuring the incorporation of theory throughout [40]. Guided by the BCW worksheets [42], physical activity was identified as the behaviour anticipated to produce the greatest reduction in CVD risk in male taxi drivers. This was due to the long periods of sedentary time associated with the occupation, the likelihood of male taxi drivers changing the behaviour, the impact that physical inactivity has on other CVD-related behaviours, and the ease of measurement in a clinical trial. Next, it was specified that male taxi drivers should aim to complete greater levels of physical activity daily, in any setting, and with whomever they wish.

The next step in the process was to ensure the intervention was grounded in behaviour change theory, beginning with the conduction of a behavioural analysis. Doing so uncovers what needs modified at the individual level for successful change to occur. Next, the outcomes of the analysis were mapped to intervention functions deemed appropriate for facilitating the necessary change. Lastly, behaviour change techniques (BCTs) that could successfully deliver the intervention functions were identified using the BCT taxonomy [41], and through an assessment of BCTs utilised in previous eHealth interventions targeting male only participants [39]. ManGuard will be delivered as an application (app), accessible through handheld devices including mobile phones and tablets.

ManGuard contains seven modules: (i) introduction and goal setting, (ii) being active (iii) eating well, (iv) managing stress, (v) smoking, (vi) alcohol, and (vii) keep accelerating, the latter to be used as a re-cap module. Each module was developed through an iterative process between the lead researcher and the PAG, prior to being refined through end-user input gathered during the focus groups. Initially, it was envisioned that participants would access modules in a sequential order, once per week, throughout the intervention period. However, end-user input recommended participants have access to the modules of their choice in any order, dependent on their preference. Therefore, the intervention period will last a total of 7 weeks in line with the number of modules available, with participants having the choice of which modules they wish to access. Each module will take no longer than 10 min to complete, with the content of each module focused on providing participants with brief and motivational information on the CVD-related behaviour or risk factor of interest. Content development was guided by published scientific recommendations, and steered by end-user input, to be delivered in a ‘tips and tricks’ format, providing short segments of text, and accompanied with images relevant to the taxi driver population. Each module uses positive and motivational language as a means of improving the likelihood of successful behaviour change.

Program features include goal setting and self-monitoring of progress [72], including physical activity, diet, weight, smoking, and alcohol. A group messaging board was envisioned as being embedded into the program, providing an opportunity for enhancing social support, shown to be effective in promoting engagement in an eHealth intervention for men [26]. However, qualitative input from end-users highlighted a social forum as being potentially problematic due to the nature of this population, the language they use, and how they interact with each other. As a result, a Question and Answer (Q&A) page will be utilised during the initial testing of ManGuard. Questions will be sent directly to the lead researcher with the answers posted publicly for all users. The questions and answers will be used as the basis of a Frequently Asked Questions page for future iterations of the program if a definitive trial is deemed feasible. The program will be interactive, with in-app motivational messages displayed as a form of automated feedback. It is envisioned that the messages will provide positive reinforcement when they are progressing well towards their goals, or as messages of encouragement if progress is poor. Participants will be provided with in-app ‘awards’, badges they collect for achieving milestones such as completing a module or making progress towards a goal. Weekly push notifications will be employed as reminders to access the program and modules, with twice-weekly notifications delivered to users as a reminder to input and track progress.

### Data collection, management, and analysis

#### Demographic characteristics

A survey will be disseminated during the participants’ first visit to the NICRF to gather demographic data. Information will pertain to the participants’ age, postcode, education level, employment status, years worked as a taxi driver, working hours, lifestyle factors, knowledge of CVD, and presence of chronic diseases diagnosed by a health professional. The information gathered form the survey will be explored to gain an understanding of factors that may influence a participant’s decision to take part in the study.

#### Feasibility measures

As this is a feasibility randomised controlled trial, the primary outcomes of interest are the following:

- Recruitment rates
- Retention rates
- Engagement with the intervention
  - Assessed by program usage via weekly analytics, including the quantity of participant logins, modules accessed, time spent on each module, and frequency of accessing the progress tracking system
- Program usability and participant satisfaction
  - Obtained as part of the process evaluation through semi-structured telephone interviews (Additional file 1) with a subset of participants. Telephone interviews will be conducted by a member of the research team who is trained and experienced in qualitative interviewing. Additionally, a satisfaction survey will be completed by all participants during the 3-month assessment time point.

#### Clinical indices

Though not powered to detect clinically significant change, the following CVD-related outcomes will be assessed to determine preliminary efficacy of ManGuard, and feasibility of each for inclusion in a definitive trial:

#### CVD biomarkers

- CVD biomarkers will be assessed through point-of-care testing with the Alere Cholestech LDX Analyzer, valid for the analysis of cholesterol and glucose measurements [10, 52]. Participants will be required to fast for 8-h prior to testing.
  - Full cholesterol breakdown (total cholesterol, HDL cholesterol, LDL cholesterol, triglycerides, and total/HDL cholesterol ratio)

#### Blood pressure

- Systolic and diastolic blood pressure will be measured using a calibrated sphygmomanometer three times during each visit, with one-minute intervals between each. An average of the three measures will be calculated and used as the final reading [62]. Measurements will be taken from a seated posiiton by a trained member of the research team, with the cuff placed in line with the midpoint of the sternum. Participants will be asked to sit upright, feet flat on the floor, for 3–5 min prior to the first measurement being taken [47].

#### Anthropometry

- BMI
- Body fat percentage will be analysed through Bioelectrical Impedance Analysis (BIA). BIA is a valid, precise, and cost-effective method of assessing body fat percentage and lean mass, both having strong correlations with mortality and morbidity [6]
- Waist circumference will be measured midway between the lowest rib and the iliac crest, proven to be the most reliable for defining central obesity [36].

#### Physical activity

Physical activity will be measured both objectively and subjectively using accelerometer data and the short-form version of the International Physical Activity Questionnaire (IPAQ-SF), respectively.

- Accelerometer data will be collected using the Actigraph GT3X + accelerometer, deemed a reliable tool for measuring physical activity in adults living in ‘free conditions’ [1]. Participants will be required to wear the accelerometer for 3–5 days to ensure the most reliable estimates of physical activity [61]
- The International Physical Activity Questionnaire (IPAQ-SF), a 7-item self-report survey of physical activity completed in the last 7 days, is a valid and reliable tool [15]. Items include frequency and intensity of physical activity, as well as time spent sedentary [15]. The IPAQ-SF has previously shown significant associations with accelerometer derived physical activity data (Murphy et al. 2017).

#### Psychosocial status

Psychosocial status will be assessed through health-related quality of life (HRQOL), self-efficacy, and social support measures.

- HRQoL will be assessed using the ‘generic’ 12-item Short Form Health Survey (SF-12) to measure physical and mental health components across the last 4 weeks [71]. The SF-12 has adequate scale reliability (Cronbach’s alpha = 0.89) and has been validated in the ‘general’ UK population [9].
- Self-efficacy will be assessed using the General Self-Efficacy Scale (GSE), a 10-item self-report measure of an individual’s self-belief in coping with a variety of difficult and demanding life tasks [34]. The GSE has demonstrated validity and reliability across 25 countries consisting of a variety of cultures and genders, with a Cronbach’s alpha of 0.88 in participants from Great Britain [56].
- Social support will be assessed using the Multidimensional Scale of Perceived Social Support (MSPSS). The MSPSS is a reliable, brief, and easy to complete [2] 12-item self-report measure of support from an individual’s family, friends and their significant other [77]. The MSPSS has strong internal reliability (Cronbach’s alpha of 0.93–0.98) in both clinical and non-clinical subjects, of different ages and gender [28].

**Fig. 1:**
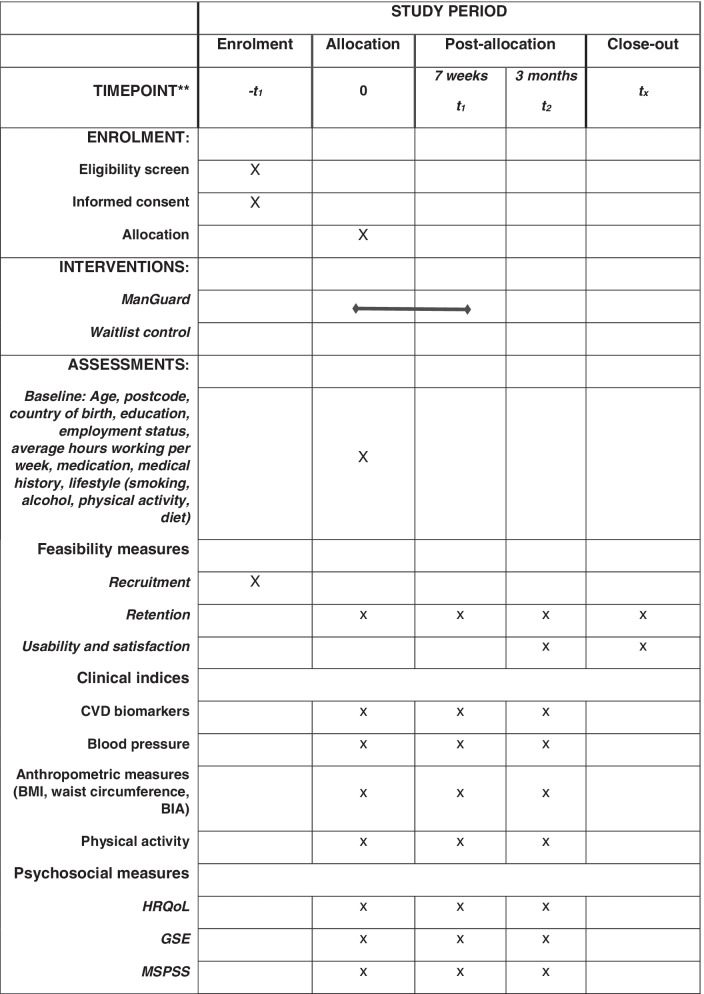
SPIRIT figure of the schedule for enrolment, intervention implementation, and assessment time points

The schedule for the enrolment or participants, administration of the intervention, and the outcomes with assessment time points is shown in Fig. 1.

#### Data management

Hard copies of data will be kept confidential and stored securely in a locked cabinet within the School of Nursing and Midwifery, Queen’s University Belfast. Soft copies of data obtained throughout the study will be stored on the Queen’s University Belfast Cloud Storage (Q) Drive.

### Statistics and data analysis

#### Quantitative analysis

Quantitative data analysis will be conducted using SPSS V.24. Descriptive statistics will be used to report on baseline demographic, clinical data, and program usability. Categorical data will be presented as frequencies and percentages, whilst continuous data is presented as means and standard deviations. Numerical data will be analysed using SPSS V.24. Repeated measures ANOVA will be used to assess the preliminary efficacy between groups across three time points for physical activity, clinical indices, and psychosocial status.

As a feasibility randomised controlled trial, and therefore not statistically powered, results will be interpreted with caution. Recruitment and retention rates will be reported on and presented using the CONSORT flow diagram [21].

#### Qualitative analysis

Semi-structured telephone interviews for collecting data on participant satisfaction, experience, and program usability will be audio recorded and transcribed verbatim. Transcripts will be analysed using conventional content analysis using an inductive approach for the coding and categorising of data. Content analysis involves a systematic approach for exploring large amounts of textual information [63], suitable for intervention refinement [49]. All participants in the intervention group will be invited to complete the telephone interviews, with interviews ceasing once data saturation is achieved [37]. Members of the research team will be involved in the qualitative analysis of data and will assess data saturation throughout the telephone interview process.

### Progression criteria

Progression to a definitive trial will be determined by the following progression criteria for participant recruitment, utilised previously in pilot and feasibility studies [11, 38].

- 75-100% of the target sample size (30) recruited to the study will warrant progression to a definitive trial
- 50-74% of the sample size (30) recruited to the study will warrant progression to a definitive trial following necessary adjustments to barriers of recruitment outlined during the process evaluation
- 25-49% sample size (30) recruited to the study will only warrant progression to a definitive trial if changes to the protocol are made by both the research team and the potential future co-applicants
- Less than 25% of the sample size (30) recruited to the study will indicate that the trial is unlikely to progress.

Progression criteria for retention rates will follow the criteria below [29]:

-  > 80% retention rate will warrant progression to a definitive trial
- 60-80% retention rate will warrant progression to definitive trial following necessary adjustments to counter attrition
-  < 60% retention rate will indicate progression to a definitive trial is unlikely

Participant satisfaction will be assessed through the process evaluation and will provide information on whether the intervention was feasible, including the process of randomisation. Progression to a definitive trial will be dependent on acceptability, with necessary modifications indicated by the participants being made prior to progressing.

## Discussion

Whilst male taxi drivers have been identified as a high-risk group of developing CVD due to their engagement in CVD-related risk behaviours, there is a dearth of literature concerning health interventions for this population. eHealth interventions may prove beneficial for the reduction in CVD risk in this population due to the ease of access and use from any location. Yet, in the absence of published research, it is unknown whether delivering such an intervention is feasible.

Previous research has identified that men benefit from their own behaviour change interventions, taking into consideration the method of engagement, the way information is presented and the mode of delivery [24]. Considering such preferences has been identified as positively influencing intervention uptake and reducing the risk of participant attrition in interventions targeting men [24]. Reducing risk of attrition is of the upmost importance in interventions with male only subjects, presenting as a common limitation in previous trials [19, 32, 58].

One such way to tailor an intervention towards men is using eHealth, providing the end-user with the ability to take an active role in their own health care [65]. Previous eHealth interventions have shown potential for promoting behaviour change and reducing CVD risk in male only participants [39]. However, trials often involve the use of an ‘active control’ [19, 25, 44, 60], which may obscure the ‘true’ perceived benefits of the eHealth intervention. This was evident whilst comparing results from these studies to those that included a ‘true control’ [43, 68], who reported greater impact from eHealth interventions on behaviour change.

Whilst research has shown male taxi drivers are at an increased risk of CVD, few studies of interventions to reduce this risk have been conducted. We aim to fill this gap by describing a protocol for a feasibility randomised controlled trial of an eHealth intervention (ManGuard) designed to reduce CVD risk in male taxi drivers. In addition, we aim to assess preliminary efficacy of ManGuard for improving clinical indices, physical activity, and psychosocial status in this high-risk group. We envisage that this study will add to the literature on the choices and preferences of men for behaviour change interventions using eHealth, whilst providing the basis for a definitive trial to assess the effectiveness of an eHealth intervention for reducing CVD risk in this high-risk and underserved population.

## Supplementary Information

**Additional file 1.** Process evaluation interview guide.

## Data Availability

Data sharing is not applicable to this article as no datasets have been generated or analysed. It is intended that data generated during the study described in this protocol will be disseminated in peer-review journals and at relevant scientific conferences.

## Abbreviations

BCTs: Behaviour Change Techniques
BIA: Bioelectrical Impedance Analysis
CVD: Cardiovascular disease
GSE: General Self-Efficacy Scale
HRQoL: Health-related quality of life
IPAQ-SF: International Physical Activity Questionnaire Short Form
MSPSS: Multidimensional Scale of Perceived Social Support
NCD: Non-communicable disease
NICRF: Northern Ireland Clinical Research Facility
NIHR: National Institute for Health Research
SF-12: 12-Item Short Form Health Survey
SPSS V.24: Statistical Package for the Social Sciences
UK: United Kingdom
