# Supplementary material for: An eHealth intervention (ManGuard) to reduce cardiovascular disease risk in male taxi drivers: protocol for a feasibility randomised controlled trial
Source: Pilot Feasibility Stud. 2022 Sep 14;8:209. doi: 10.1186/s40814-022-01163-4 (PMC9472349; doi:10.1186/s40814-022-01163-4)
Supplement: Supplementary file 1 — Additional file 1. Process evaluation interview guide. [file 40814_2022_1163_MOESM1_ESM.docx]

**Process evaluation interview guide**

**Opening conversation**

Hello, thank you for taking time out of your day and agreeing to speak to me. In this interview I want to get your opinion on the ManGuard program that you completed to see what you thought of it and whether it can be improved.

If you would like to take a break or stop at any time, please let me know. I would like to reassure you of the confidentiality of this research, and that your name will not be attached to any transcripts following this interview or attached to any report that is produced and published.

Do you have any questions you would like to ask before we begin the interview?

I would like to remind you that this interview will be audio recorded before being transcribed, just to check that this is okay with you?

Shall we begin?

**ManGuard program**

1. What was your general opinion of ManGuard?
2. What was your opinion on the length of the program?

*Would they have preferred it to be delivered over a different length of time? If so, what would suit them best and why?*

1. What was your opinion on the number of modules to complete per week?

*Would they have preferred the modules to be completed more regularly than once a week? If so, how many per week and why?*

1. What did you think about the content of the modules?

*Did it make them more aware of heart disease and the associated risks?*

*What did they feel was the most and least helpful information? anything not covered that they would have liked to have been included?*

1. Before beginning the program, you were recommended to set yourself a goal that you would like to achieve by the end of the program. Did you set a goal?
2. If you did set a goal, what was it and did the program help you to achieve this?
3. Did the program result in any positive changes to your lifestyle?

*If answer is yes, ask what they changed.*

*If answer is no, ask them why? And if there was any extra information that could have been included that would have made them more motivated to make change.*

1. Did you find accessing the ManGuard program easy?

*Expand on what they liked, disliked and what could be approved.*

*Probe on their opinion of functionality, accessibility, and the ease of navigating around the different modules.*

1. What was the best thing about ManGuard (if there was one)?
2. What was the worst thing about ManGuard (if there was one)?
3. Are there any changes that you feel could be made to the ManGuard program that would increase the likelihood of other drivers engaging with it?
4. Are there any additionally comments you would like to make in regard to ManGuard?

I would just like to thank you once again for taking time to answer my questions. Is there anything at all you would like to ask before we end this interview?
